# Long-Term Survival of Human Enteric Virus Surrogates (Murine Norovirus, Rhesus Rotavirus, and Porcine Sapovirus) in Bottled Drinking Water

**DOI:** 10.1007/s12560-026-09710-5

**Published:** 2026-09-24

**Authors:** Ziwei Zhao, Leena Maunula

**Affiliations:** Department of Food Hygiene and Environmental Health, Faculty of Veterinary Medicine, University of Helsinki, Helsinki, Finland

**Keywords:** Murine norovirus, Rhesus rotavirus, Porcine sapovirus, Bottled drinking water, Viral persistence, Environmental stability

## Abstract

Enteric viruses are major causes of food- and waterborne diseases. Due to the limited cultivability of many human enteric viruses, surrogate viruses are commonly used to model their environmental persistence. This study aimed to evaluate the not-well-understood long-term survival of murine norovirus (MNV), rhesus rotavirus (RRV), and porcine sapovirus (PoSaV), used here as cultivable surrogate viruses for human enteric viruses, in bottled drinking water. Bottled water artificially inoculated with each virus was stored at 4 °C, 25 °C, and 37 °C for up to 365 days, and viral infectivity was quantified using TCID_50_ and plaque assays, with the latter successfully applied for PoSaV quantification. The initial virus concentrations were 5.2 log_10_ TCID_50_/mL for MNV, 6.5 log_10_ PFU/mL for RRV, and 4.0 log_10_ PFU/mL for PoSaV. MNV and RRV remained infectious for at least one year at 4 °C, whereas PoSaV gradually declined and became undetectable by day 55. At 25 °C, the viral inactivation rate was accelerated: MNV became undetectable by day 160, RRV by day 320, and PoSaV by day 20. At 37 °C, a rapid inactivation rate was observed for all viruses, with MNV, RRV, and PoSaV becoming undetectable by days 20, 80, and 10, respectively. These results demonstrate the pronounced temperature-dependent survival of enteric viruses in bottled water. It is important to ensure the use of microbiologically clean source for bottled water as well as implement effective food and water safety management to minimise potential viral contamination.

## Highlights

The long-term survival of MNV, RRV, and PoSaV in bottled drinking water was evaluated.

The viral inactivation rate increased with temperature (25–37 °C).

MNV and RRV remained infectious for at least 365 days at 4 °C.

In our study, PoSaV demonstrated lower stability than both MNV and RRV.

Enteric viruses are extremely persistent in bottled water at low temperatures.

## Introduction

The World Health Organization estimates that unsafe food leads to approximately 600 million foodborne illnesses and 420,000 deaths each year (WHO, 2024). Foodborne enteric viruses such as norovirus, rotavirus, and sapovirus cause substantial morbidity worldwide. Enteric viruses transmitted via the faecal–oral route are typically highly infectious, environmentally stable, and shed in large quantities (Robilotti et al., 2015; Vinjé, 2015). Their transmission occurs through multiple pathways, including direct person-to-person spread and indirect spread through contaminated food, water, and fomites (Robilotti et al., 2015; Vinjé, 2015). In the United States, norovirus caused approximately 5.5 million of the ~ 9.9 million domestically acquired foodborne illnesses attributed to seven major pathogens in 2019 (≈ 56%) (Scallan Walter et al., 2025). In the European Union, norovirus (and other caliciviruses) was a leading cause of foodborne outbreaks in 2024 (*N* = 14,297) across Member States (EFSA and ECDC, 2025). Bottled water is legally classified as a food product and is therefore subject to food safety legislation and microbiological quality standards rather than drinking water regulations (Department for Environment, Food & Rural Affairs, 2015). Because important human enteric viruses such as human norovirus and sapovirus remain difficult to cultivate routinely, experimentally tractable surrogate viruses are widely used to study viral persistence and inactivation in food and environmental systems (Arthur & Gibson, 2015; Bozkurt et al., 2015).

Among foodborne viruses, human norovirus (HuNoV), which infects persons of all ages, is the dominant cause of acute non-bacterial gastroenteritis outbreaks worldwide (Robilotti et al., 2015). Norovirus alone, including person-to-person transmission, is estimated to cause approximately 685 million cases and ~ 200,000 deaths annually (WHO, 2022). Noroviruses are small (~ 27–40 nm), non-enveloped, positive-sense single-stranded RNA viruses within the family *Caliciviridae*. They are genetically diverse and divided into multiple genogroups, several of which infect humans (Vinjé, 2015; De Graaf et al., 2016; Chhabra et al., 2024). Indirect evidence of norovirus infectivity in environmental matrices has been demonstrated during waterborne outbreaks, such as the bottled mineral water-associated outbreak in Spain, where norovirus GI and GII were detected (Guix et al., 2020). Recent advances in human intestinal enteroid systems have enabled the cultivation of human norovirus under laboratory conditions (Ettayebi et al., 2016). However, these technically demanding systems are not yet widely available. Consequently, cultivable surrogates such as MNV and Tulane virus are still widely used to investigate human norovirus infectivity, persistence, and inactivation in food and environmental matrices (Robilotti et al., 2015).

Although rotaviruses are less frequently implicated in foodborne outbreaks than noroviruses, they remain an important cause of acute gastroenteritis, particularly among infants and young children worldwide (Troeger et al., 2018). Waterborne outbreaks associated with rotavirus contamination have also been reported, including an outbreak linked to contaminated water of refilling stations in the Philippines in 2016 (Rebato et al., 2019; Kukkula et al., 1999). Rotaviruses are ~ 75 nm (in diameter), non-enveloped viruses with a distinctive triple-layered icosahedral capsid and an 11-segmented double-stranded RNA genome, belonging to the family *Sedoreoviridae* (Trask et al., 2012). This multilayered capsid structure confers marked environmental stability and resistance to physicochemical stress, facilitating persistence in the environment (Desselberger, 2014). Following the introduction of rotavirus vaccination programmes, substantial declines in rotavirus-associated hospitalisations and severe gastroenteritis have been observed in many countries (Tate & Parashar, 2014; Burnett et al., 2020). Human infections are mostly caused by group A rotaviruses, which are primarily classified into G and P genotypes based on the outer capsid proteins VP7 and VP4, with extended classification based on all 11 genomic RNA segments (Matthijnssens et al., 2008). Although human rotaviruses can be cultivated in cell lines, viral titres are often low; therefore, rhesus rotavirus (RRV) or simian rotavirus strain SA-11 is frequently used as a surrogate in experimental studies (Trask et al., 2012).

Sapoviruses are less frequently reported than noroviruses and rotaviruses but cause acute gastroenteritis, predominantly in children but also affecting adults worldwide (Oka et al., 2015; Liu et al., 2016). Sapoviruses are small (~ 30–38 nm), non-enveloped, positive-sense ssRNA viruses belonging to the family *Caliciviridae*, like noroviruses (Oka et al., 2015). Sapoviruses have been associated with food- and waterborne infections as well as sporadic cases and outbreaks of gastroenteritis, including drinking water outbreaks linked to wastewater intrusion. In Finland, several sapovirus-associated waterborne outbreaks have been reported, including one outbreak described by Jalava et al. (2019) and two outbreaks reported by Kauppinen et al. (2019). Like norovirus, sapoviruses are also classified into multiple genogroups and genotypes, among which GI and GII are most frequently detected in human gastroenteritis cases worldwide, whereas other genogroups circulate predominantly in animals such as pigs (Reuter et al., 2010; Tang et al., 2022). Porcine sapoviruses (PoSaV) are used as surrogates because the Cowden strain can be cultured in vitro in the presence of bile acids, whereas human sapoviruses have remained difficult to cultivate (Hosmillo et al., 2014) until these days. Despite increasing detection of sapoviruses in environmental waters and food matrices, data on their persistence in water remain limited and it is less studied than those of noroviruses (Kitajima et al., 2018).

More information on the long-term persistence of infectious enteric viruses in bottled water is still required, particularly comparative studies using cultivable viruses over extended time periods lasting several months. Bottled water is often stored for prolonged periods under variable temperature conditions and is widely consumed by vulnerable populations. Therefore, the objective of this study was to investigate the long-term survival of MNV, RRV, and PoSaV in commercially bottled drinking water stored at 4 °C, 25 °C, and 37 °C. The selected temperatures represent refrigeration, ambient storage, and elevated temperature conditions that may occur during storage, transportation, and retail display of bottled water. Although the persistence of MNV has previously been investigated in various environmental and water matrices (Zhao et al., 2022; Wu et al., 2023; Girón-Guzmán et al., 2025), MNV was included in the present study to enable direct comparison of its persistence with those of RRV and PoSaV under identical experimental conditions.

## Materials and Methods

### Viruses and Cell Culture

MNV (MNV-1; supplied by Prof. Herbert W. Virgin IV, University of Washington, USA) was cultivated in RAW 264.7 γNO(−) cells (mouse monocyte-macrophage line, ATCC^®^ CRL-2278™). The cells were cultured in Dulbecco’s Modified Eagle Medium (DMEM; Gibco™, 42430025) with high glucose and HEPES, supplemented with 10% heat-inactivated fetal bovine serum (FBS; Gibco™, 10500064) and 1% L-Glutamine–Penicillin–Streptomycin (GPS; Sigma-Aldrich, G1146). Cells were maintained at 37 °C in a humidified 5% CO_2_ incubator. After cultivation, virus stocks were prepared by three freeze–thaw cycles, followed by low-speed centrifugation (300 × g at 4 °C) to remove cell debris, as described in Loikkanen et al. (2025). Prior to the survival experiments, FBS was removed from the virus suspension to avoid potential interference from serum components with viral stability. The virus-containing culture medium was subjected to ultrafiltration using Amicon^®^ Ultra centrifugal filter units (100 kDa MWCO; Millipore, UFC9100) at 3200 × g for 10 min at 15 °C. Subsequently, the retentate was brought back to the original volume with Phosphate-Buffered Saline (PBS, pH = 7.4) to minimise changes in viral concentration, and the post-exchange titre was not re-determined.

RRV, kindly provided by Dr. Lennart Svensson (Linköping University, Sweden), was propagated in MA-104 cells (rhesus monkey kidney epithelial cells, ATCC^®^ CRL-2378.1), as described in the rotavirus protocol by Arnold et al. (2009). Cells were cultured in Eagle’s Minimum Essential Medium (EMEM; Sigma-Aldrich, M2279) supplemented with 10% FBS and 1% GPS. Prior to infection, the virus was activated with 10 µg/mL of trypsin (Trypsin from porcine pancreas; Sigma-Aldrich, T0303) for 1 h at 37 °C. After adsorption, cells were overlaid with serum-free EMEM (0% EMEM) containing 1 µg/mL trypsin. Virus stocks were prepared by three freeze–thaw cycles, followed by low-speed centrifugation (300 × g at 4 °C) to remove cell debris. Amicon treatment was not performed, as the virus was propagated in serum-free EMEM.

PoSaV (Cowden strain) was cultivated in LLC-PK1 cells (ATCC^®^ CL-101), a porcine kidney epithelial cell line. The PoSaV strain used in this study was provided by Prof. Dr. Linda J. Saif from Ohio State University, USA. Cells were maintained in EMEM supplemented with 5% FBS, 1% non-essential amino acids (NEAA; Gibco™, 11140035), and 1% GPS. For virus infection and replication, the culture medium was replaced with serum-free EMEM supplemented with 100 µM glycochenodeoxycholic acid (GCDCA; Avanti Polar Lipids, 700266P), a bile acid shown to promote calicivirus replication. Cells were incubated at 37 °C with 5% CO₂ and monitored for cytopathic effects (CPE). Virus stocks were prepared by three freeze–thaw cycles, followed by low-speed centrifugation (300 × g at 4 °C) to remove cell debris. The clarified virus suspension was further concentrated 10-fold to increase the virus titre using Amicon^®^ Ultra centrifugal filter units without medium exchange, since it was cultivated in the presence of serum-free medium.

###  Virus Inoculation into Bottled Drinking Water

Commercial bottled drinking water (spring water, Polar Spring, Finland) was purchased from a Finnish supermarket. The chemical composition and pH of the water are summarised in Table 1. The titres of the propagated virus stocks were the following, as determined before Amicon treatment: MNV at an initial concentration of 7.6 log_10_ TCID_50_/mL, RRV at 7.9 log_10_ PFU/mL, and PoSaV at 4.7 log_10_ PFU/mL.

**Table 1:** Chemical composition of the commercial bottled drinking water (Polar Spring, Finland) used for virus survival assays (according to the label)

Component	Concentration
Calcium (Ca)	12 mg/L
Magnesium (Mg)	4.5 mg/L
Sodium (Na)	5.3 mg/L
Potassium (K)	2.4 mg/L
Fluoride (F)	< 0.10 mg/L
Nitrate (NO_3_)	3.8 mg/L
pH	6.6
Evaporation residue (180 °C)	99 mg/L

For MNV and RRV, 12 mL of virus suspension was added to bottled water to obtain a final volume of 600 mL. The mixture was gently inverted and allowed to stand for 30 min to ensure homogeneous distribution of the virus in the water matrix. The inoculated water was then aliquoted into thirty sterile 50 mL conical tubes, each containing 20 mL of virus-inoculated water. The aliquoted samples were assigned to three storage temperatures (4 °C, 25 °C, and 37 °C), with ten replicate tubes per temperature. At each sampling time point (0, 5, 10, 20, 40, 80, 160, 240, 320, and 365 days post-inoculation), one tube from each temperature was removed for virus infectivity quantification.

For PoSaV, because of the lower virus titre, a modified inoculation procedure was used. Briefly, 3 mL of the PoSaV suspension concentrated 10-fold using Amicon^®^ Ultra centrifugal filter units was mixed with 27 mL of bottled water to obtain a total volume of 30 mL. The mixture was gently mixed and divided into three sterile tubes containing 10 mL each, which were incubated separately at 4 °C, 25 °C, and 37 °C. At each sampling time point, 1 mL was withdrawn from each tube for virus infectivity quantification.

All samples were stored in the dark to avoid photoinactivation. Following infectivity quantification, the remaining sample was transferred to sterile tubes and stored at − 72 °C as a backup. All procedures were performed under aseptic conditions in a Class II biosafety cabinet, and each virus was tested independently.

### Virus Quantification Methods

MNV infectivity was quantified using the median tissue culture infectious dose (TCID_50_) assay, following the method described by Mosselhy et al. (2022). Briefly, 100 µL of each 10-fold serially diluted water sample was added to six parallel wells of confluent RAW 264.7 γNO(−) cell monolayers (at ~ 90–100% confluence) in 96-well plates (Nunc™ MicroWell™ 96-well, Thermo Scientific™, 161093). After virus inoculation, plates were incubated for 1 h at 37 °C in 5% CO_2_ to allow adsorption. The inoculum was then removed and replaced with 200 µL of 10% DMEM per well (i.e., DMEM supplemented with 10% FBS and 1% antibiotics). Plates were incubated for five days under standard cell culture conditions (37 °C, 5% CO_2_). The presence of viral infection was determined by observing CPE under an inverted light microscope. Viral titres were calculated using the Reed-Muench method and expressed as log_10_ TCID_50_/mL (Reed & Muench, 1938; Lei et al., 2021). The LOD was estimated based on the assay conditions (100 µL per well, six replicate wells, starting dilution of 10^− 1^). Under these conditions, the analytical LOD was 5 TCID_50_/mL (0.7 log_10_ TCID_50_/mL) when assuming one infectious unit as the detection threshold.

RRV was quantified by plaque assay using MA-104 cells, following a protocol adapted from Arnold et al. (2009). MA-104 cells were seeded into 12-well plates (Costar^®^ 12-well Clear TC-treated Multiple Well Plates, Corning, 3512) at 1 mL per well and incubated for 4 days to reach confluence (at ~ 90–100% confluence). Rotavirus in the water samples were activated by incubation with trypsin (final concentration of 10 µg/mL) in a 37 °C water bath for 1 h. Ten-fold serial dilutions were then prepared in 0% EMEM. Prior to inoculation, MA-104 monolayers were washed three times with a prewarmed 0% EMEM. Then, 100 µL of each virus dilution was added to duplicate wells and incubated at 37 °C for 1 h. After adsorption, the inoculum was removed, and cells were washed once with 0% EMEM.

The overlay medium was prepared by mixing one part of molten 3% (w/v) sterile agarose with nine parts of prewarmed 0% EMEM. Trypsin was subsequently added to achieve a final concentration of 0.75 µg/mL. After ensuring the temperature of the overlay was below 42 °C, 1 mL overlay was gently added to each well. Plates were incubated at 37 °C with 5% CO_2_ for 3–4 days, until plaques became visible. Cells were fixed with 1 mL of 3.7% formaldehyde per well for at least 4 h at room temperature (RT), rinsed with tap water to remove the overlay, and stained with 0.1% crystal violet (Merck, C.I. 42555) for 10 min. Plaques were counted manually, and viral titres were expressed as log_10_ plaque-forming units per milliliter (PFU/mL). The LOD for RRV was calculated based on the plaque assay conditions (100 µL per well, duplicate wells, starting dilution of 10⁻¹). Under these conditions, the LOD was estimated to be 0.7 log_10_ PFU/mL assuming a threshold of 1 PFU.

PoSaV infectivity was evaluated using a plaque assay. LLC-PK1 cells in 5% EMEM were seeded into 12-well plates at 1 mL per well and incubated for 5 days to reach confluence (at ~ 90–100% confluence). Virus samples were serially ten-fold diluted in serum-free medium and inoculated onto the cell monolayers (200 µL per well) without prior washing, to minimise cell detachment and maintain monolayer integrity during infection. Plates were incubated at 37 °C for 1 h with gentle rocking every 15 min to allow virus adsorption.

Following adsorption, the inoculum was removed, and cells were overlaid with 1 mL of overlay medium, as for RRV. The procedure was otherwise identical, with the exception that GCDCA was added to the medium at a final concentration of 100 µM prior to the addition of agarose. The mixture was gently mixed by inversion to ensure homogeneity before application to the cells. Plates were incubated at 37 °C in a 5% CO_2_ incubator for 3 days until visible plaques were formed. After incubation, fixation, overlay removal, and staining were performed as described above for RRV. Plaques were counted, and virus titres were calculated as PFU/mL. The LOD was estimated based on the plaque assay conditions (200 µL per well, duplicate wells, starting dilution of 10⁻¹), assuming one plaque as the detection threshold. Under these conditions, the analytical LOD was approximately 25 PFU/mL (1.4 log_10_ PFU/mL).

Each assay included mock-infected negative controls and standard virus dilutions as positive controls. All experiments were performed in triplicates.

### Statistical Analysis

For each condition, three plates with duplicate wells were used, resulting in six technical replicates per experiment. The experiments were performed independently twice. Data are presented as means ± standard deviation (SD). Viral titres were expressed as log_10_ TCID_50_/mL (MNV) or log_10_ PFU/mL (RRV, PoSaV). All statistical analyses and significance plotting, including multiple pairwise comparisons (t tests with appropriate correction for multiple testing), were performed using GraphPad Prism version 10.6.0 (GraphPad Software, USA). Statistical significance in the figures is indicated as follows: * *P* < 0.05, ** *P* < 0.01, *** *P* < 0.001, and ns = not significant.

## Results

### Survival of Murine Norovirus in Bottled Drinking Water

**Fig. 1:**
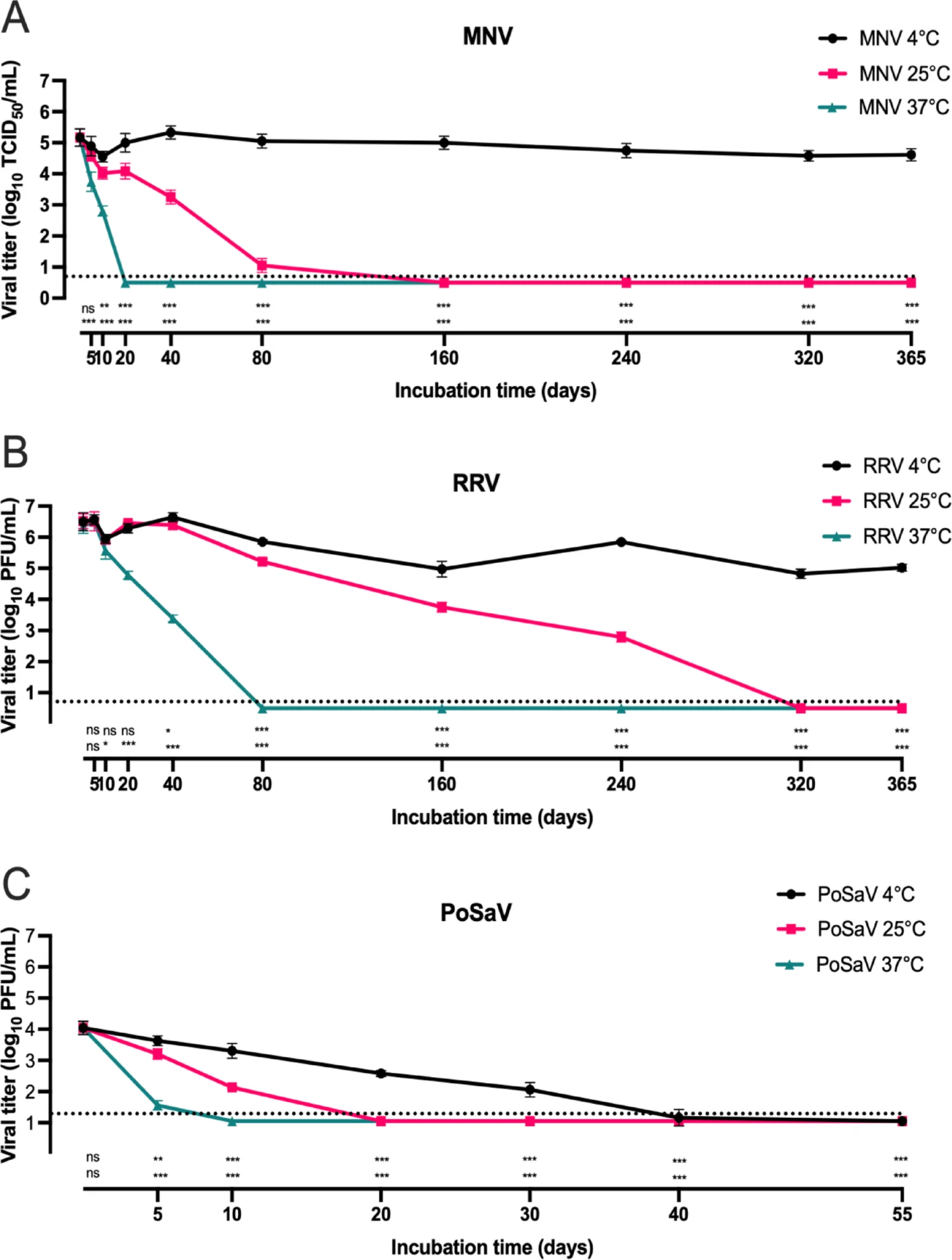
Survival of murine norovirus (MNV) (**A**), simian rotavirus (RRV) (**B**), and porcine sapovirus (PoSaV) (**C**) in bottled water during storage at 4 °C, 25 °C, and 37 °C, as determined using TCID_50_ assay for MNV and plaque assay for RRV and PoSaV. MNV and RRV were monitored for one year, whereas PoSaV was monitored for 55 days. The dashed line represents the limit of detection (LOD). Data are presented as mean ± standard deviation (SD). Significance was assessed by multiple unpaired t-tests against the 4 °C control at each time point. In the figure, the upper row of significance markers corresponds to 25 °C vs. 4 °C, and the lower row corresponds to 37 °C vs. 4 °C. ***P < 0.001, **P < 0.01, *P < 0.05, and *ns*  not significant

The survival kinetics of MNV in bottled water is shown in Fig. 1A. The initial recovery titre of MNV was 5.2 ± 0.3 log_10_ TCID_50_/mL at day 0. At 4 °C, MNV remained infectious throughout the 365-day storage period, with a final titre of 4.6 ± 0.1 log_10_ TCID_50_/mL at day 365, which was significantly lower than that at day 0 (unpaired t-test, adjusted P = 0.0058). A transient decrease in viral titre was observed at day 10 (4.6 ± 0.2 log_10_ TCID_50_/mL), which was also significantly lower than that at day 0 (unpaired t-test,adjusted *P* = 0.0016). However, titres recovered at subsequent sampling points, suggesting that this early decrease was likely due to technical variation.

At 25 °C, MNV titres gradually decreased to 3.2 ± 0.2 log_10_ TCID_50_/mL at day 40 and 1.1 ± 0.2 log_10_ TCID_50_/mL at day 80. Infectious virus levels were below the LOD at day 160 and remained there through day 365. At 37 °C, a rapid decline in MNV titre was observed, with titres dropping to 3.7 ± 0.3 log_10_ TCID_50_/mL by day 5 and 2.8 ± 0.2 log_10_ TCID_50_/mL by day 10. No infectious MNV was detected from day 20 onward.

According to the pairwise statistical comparisons (summarized in Fig. 1A), MNV showed no significant reduction at 25 °C on day 5, but titres became significantly lower than the 4 °C control at day 10 and showed highly significant reductions from day 20 onward (multiple unpaired t-tests, *P* < 0.001). At 37 °C, a highly significant loss of infectivity (multiple unpaired t-tests, *P* < 0.001) was detected as early as day 5 and this loss persisted at all subsequent time points until the virus became undetectable.

### Survival of Simian Rotavirus in Bottled Drinking Water

The survival kinetics of RRV in bottled water is shown in Fig. 1B. The initial recovery titre of RRV was 6.5 ± 0.3 log_10_ PFU/mL at day 0. At 4 °C, RRV remained infectious throughout the 365-day storage period. Viral titres were 5.8 ± 0.1 log_10_ PFU/mL at day 240 and 5.0 ± 0.1 log_10_ PFU/mL at day 365, both of which were significantly lower than the titre at day 0 (unpaired t-test, adjusted *P* < 0.0001). A transient decrease in viral titre was observed at day 10 (5.9 ± 0.1 log_10_ PFU/mL), but titres subsequently recovered at days 20–240 to levels comparable to those observed before the decrease.

At 25 °C, RRV titres decreased gradually to 6.4 ± 0.1 log_10_ PFU/mL at day 40, 3.8 ± 0.2 log_10_ PFU/mL at day 160, and 2.8 ± 0.2 log_10_ PFU/mL at day 240. After 320 days, RRV titre was below the LOD. At 37 °C, a faster decline rate was observed, with titres falling to 4.8 ± 0.1 log_10_ PFU/mL by day 20 and 3.4 ± 0.1 log_10_ PFU/mL by day 40, and no infectious RRV was recovered by day 80. In this study, RRV remained infectious for a prolonged period at 4 °C and 25 °C and survived longer than MNV under the same conditions.

Based on the statistical comparisons with 4 °C as control, summarized in Fig. 1B, no significant reduction in RRV titre was detected at 25 °C up to day 20, but statistical significance was detected at day 40 (multiple unpaired t-tests, *P* < 0.05) and was highly significant (*P* < 0.001) from day 80 through day 365. At 37 °C, a significant reduction was detected at day 10 (multiple unpaired t-tests, *P* < 0.05) and highly significant loss of infectivity was observed from day 20 onward (*P* < 0.001), remaining consistent until no viable virus was detectable.

### Survival of Porcine Sapovirus in Bottled Drinking Water

The survival kinetics of PoSaV in bottled water is shown in Fig. 1C. The initial recovery titre of PoSaV was 4.0 ± 0.2 log_10_ PFU/mL at day 0. At 4 °C, PoSaV titres gradually decreased over time to 3.6 ± 0.2 log_10_ PFU/mL at day 5, 3.3 ± 0.2 log_10_ PFU/mL at day 10, and 2.6 ± 0.1 log_10_ PFU/mL at day 20. Further reductions were observed to 2.1 ± 0.2 log_10_ PFU/mL at day 30 and 1.4 ± 0.1 log_10_ PFU/mL at day 40, with viruses remaining undetectable (LOD 1.4 log_10_ PFU/mL) by day 55.

At 25 °C, a faster inactivation rate was observed. The titre decreased to 3.2 ± 0.2 log_10_ PFU/mL at day 5 and 2.1 ± 0.0 log_10_ PFU/mL at day 10, falling below the LOD by day 20. At 37 °C, PoSaV infectivity titres declined most rapidly. The titre dropped to 1.5 ± 0.2 log_10_ PFU/mL at day 5 and was undetectable at day 10.

Based on the statistical comparisons summarized in Fig. 1C, PoSaV titres at 25 °C showed a rapid decline compared with the 4 °C control. A statistically significant reduction was already observed at day 5 (multiple unpaired t-tests, *P* < 0.05), and titres were highly significantly lower from day 10 onward (*P* < 0.001). At 37 °C, PoSaV infectivity titres decreased even more rapidly. A highly significant reduction (multiple unpaired t-tests, *P* < 0.001) compared with the 4 °C control was detected as early as day 5, and titres remained under the LOD from day 10 onward.

### Comparative Analysis

When comparing the persistence of MNV, RRV, and PoSaV under identical storage conditions, clear temperature-dependent survival patterns were observed. At 4 °C, MNV and RRV maintained infectivity over the 12-month period, although infectivity titres at day 365 were significantly lower than those at day 0. In contrast, PoSaV infectivity showed a gradual decline, and was undetectable at day 55. At 25 °C, all three viruses showed reduced persistence, with RRV, MNV, and PoSaV titres falling below the LOD by days 320, 160, and 20, respectively. At 37 °C, viral inactivation rate was markedly accelerated, with RRV, MNV, and PoSaV being undetectable by days 80, 20, and 10, respectively. In both cases, the order of RRV, MNV, and PoSaV reflects their relative stability, from highest to lowest.

## Discussion

Recent studies have investigated enteric virus persistence in environmental waters, including water microcosms and irrigation water (Girón-Guzmán et al., 2025; Wu et al., 2023). In contrast, the present study focused on generating long-term infectivity data in bottled drinking water and included porcine sapovirus, for which infectivity-based persistence data in water remain limited. This study demonstrates that human enteric virus surrogates can remain infectious in commercially bottled water for extended periods, particularly under refrigeration. Temperature was found to be the dominant factor affecting viral persistence. MNV and RRV remained infectious at 4 °C for one year, whereas PoSaV with lower titre was detectable less than two months. At higher temperature, MNV was less persistent than RRV, and PoSaV became undetectable within days to weeks. These long-term infectivity data under storage-relevant conditions provide quantitative evidence that bottled water may support prolonged survival of infectious viruses if contamination occurs.

In the present study, MNV showed pronounced temperature-dependent persistence in bottled water. At 4 °C, it remained infectious for at least one year, with a decrease of less than 1 log_10_ TCID_50_/mL in titre over the 365-day storage period. This is broadly consistent with Zhao et al. (2022), who likewise demonstrated long-term persistence of MNV in bottled water at 4 °C, although their unit was PFU/mL, whereas ours was TCID_50_/mL. In contrast, more rapid reductions in MNV load at 4 °C have been reported in other aqueous matrices: Leblanc et al. (2019) observed a > 2.5 log_10_ PFU/mL reduction in spring water over 21 days (while MNV remained stable in DMEM at 4 °C), and Zhu et al. (2020) reported a 2.5-log_10_ TCID_50_/mL reduction of MNV in surface waters at 4 °C over 42 days. These findings suggest that, even at the same low temperature, the magnitude of infectivity loss depends strongly on the suspension matrix, initial virus titre, and experimental conditions. Differences among studies may be partly explained by methodological factors. For example, Leblanc et al. (2019) filtered 250 mL of water samples, which may have resulted in virus loss during processing, whereas Zhu et al. (2020) used purified recombinant MNV-1. In contrast, the present study employed less processed virus preparations, including an ultrafiltration step that may have better preserved viral integrity.

At ambient temperature, MNV infectivity titres declined progressively at 25 °C, with the virus last detected on day 80, but not on day 160. Zhao et al. (2022) reported a > 6.4 log_10_ PFU/mL reduction at 20 °C within 160 days. Despite differences in storage temperature (20 vs. 25 °C) and infectivity units (PFU vs. TCID_50_), both studies show comparable time-to-LOD under RT conditions. Supporting more rapid decay in environmental waters, Leblanc et al. (2019) reported > 2.9 log_10_ PFU/mL MNV reduction in spring water and a > 3.9 log_10_ PFU/mL MNV reduction in DMEM at 21 °C within 21 days, and Zhu et al. (2020) observed MNV titre approaching the LOD at 22 °C within 42 days, which was the final sampling point in their study. Loikkanen et al. (2025) reported an approximately 1.8 log_10_ TCID_50_/mL reduction in MNV titre over 28 days at RT, illustrating moderate decay in a cell culture medium, and emphasizing matrix-dependent kinetics at comparable temperatures. In the present study, the MNV reduction by day 20 was approximately 1.1 log_10_ TCID_50_/mL, which is slightly lower than that reported by Loikkanen et al. (2025) over a similar time frame, indicating broadly comparable decay rates under these conditions.

At elevated temperatures, a rapid rate of MNV inactivation was observed at 37 °C, with infectivity titres falling below the detection limit by day 20; similarly, Zhao et al. (2022) reported a > 6.4 log_10_ reduction within 20 days at 35 °C. Elmahdy et al. (2018) demonstrated that MNV decay in surface water microcosms is influenced not only by temperature but also by environmental factors such as light exposure, with a faster inactivation rate observed under sunlight. In the present study, samples were stored in the dark, thereby excluding photoinactivation and allowing temperature effects to be assessed independently.

HuNoV replication studies in human intestinal enteroid (HIE) systems (Ettayebi et al., 2016) suggest that infectious HuNoV can persist in water for days to weeks, depending on temperature and matrix composition. For example, Desdouits et al. (2022) demonstrated that two HuNoV strains survived for several weeks in seawater. Schaffer et al. (2022) reported that a single replicate survived for 28 days in surface water. Recently, Rexin et al. (2024) exhibited that infectious HuNoV remained detectable for up to approximately 21 days in estuarine water at 4 °C and 16 °C. Esseili et al. (2025) determined NoV persistence in freshwater between one and seven days. The present study demonstrated that the surrogate MNV remained infectious for at least one year at 4 °C in bottled water. Differences in results are likely due to the use of a surrogate or human virus, various detection methods, and water matrices. In the future, conducting survival studies that compare the survival rates of MNV and HuNoV would be highly valuable.

At 4 °C, RRV remained infectious in bottled water throughout the 365-day storage period, although infectivity decreased significantly over time. This persistence under refrigerated conditions is consistent with previous studies demonstrating that rotaviruses can survive for extended periods in water environments at low temperatures. For example, Raphael et al. (1985) reported that human rotavirus load in raw water decreased by approximately 2 log_10_ over 64 days at 4 °C, while in tap water and filtered water, the viral titre showed little or no significant decline over the same period. Similarly, Sattar et al. (1984) observed that SA-11 load in tap water stored in the dark exhibited only a limited reduction of about 0.7 log_10_ PFU over 64 days at 4 °C. In bottled water specifically, Leblanc et al. (2019) found no significant drop in bovine rotavirus titre over 21 days at 4 °C, further supporting the high stability of rotaviruses in this type of water matrix. In addition, Espinosa et al. (2008) demonstrated that RRV titre remained at approximately the same infectivity level for up to seven months in groundwater at 15 °C. Together, these studies and our results indicate that rotaviruses can persist for long periods in aquatic environments under low-temperature conditions.

At 25 °C, RRV infectivity gradually declined over time in bottled water and remained detectable until day 240, after which no infectious virus was recovered. This pattern indicates that moderate temperatures accelerate viral decay compared with refrigerated conditions but still allow for long-term persistence. Previous studies have also reported faster rotavirus inactivation at ambient temperatures. Likewise, Sattar et al. (1984) reported a 2 log_10_ PFU reduction of SA-11 in tap water over 64 days at 20 °C, indicating an increased inactivation rate at higher temperatures. Environmental water studies further support the influence of temperature and water matrix on rotavirus persistence. Espinosa et al. (2008) found that RRV declined by approximately 4 log units in surface water at RT over 210 days, reaching near the LOD, whereas the virus remained even more stable in groundwater. In addition, Leblanc et al. (2019) reported no significant reduction in bovine rotavirus titre in bottled water over 21 days at 21 °C, suggesting that the specific water matrix can strongly influence virus stability. Overall, the gradual decline of RRV load observed in the present study at 25 °C aligns with previous findings that rotaviruses remain relatively persistent in water at moderate temperatures, although their infectivity titres decrease more rapidly than under refrigerated storage.

At 37 °C, RRV infectivity titre declined rapidly in bottled water, with no infectious virus detected after approximately 80 days. This confirms that elevated temperatures markedly accelerate the rotavirus inactivation rate compared with 4 °C and 25 °C. Data on rotavirus survival rates at temperatures near 37 °C remain limited in the literature. Davidson et al. (2013) demonstrated that the porcine rotavirus OSU strain survived longer at 4 °C than at both 25 °C and 37 °C. However, since the virus persistence was studied in soil, the survival rates cannot be directly compared. Together, these findings indicate that while rotavirus can persist for prolonged periods at low and moderate temperatures, its survival rate is substantially reduced under warmer conditions.

In contrast to MNV and RRV, PoSaV exhibited markedly lower stability in bottled drinking water in the present study, with a rapid loss of infectivity observed across all temperature conditions. Even at 4 °C, PoSaV titres fell below the LOD in less than two months, whereas at 25 °C and 37 °C, the infectious virus survived for approximately two weeks and less than ten days, respectively. These results suggest that PoSaV is less stable in water compared with the other enteric viruses examined in this study. The observed rapid decline in PoSaV titre may be partly influenced by factors such as the lower initial virus titre, differences in virus stock preparation, and the specific cell culture conditions required for PoSaV propagation, including the use of bile acids.

To date, infectivity-based data on sapovirus persistence in water remain scarce, with most studies focusing on environmental detection or outbreak investigations rather than survival measurements (Kitajima et al., 2018; Kauppinen et al., 2019). Experimental evidence is largely limited to food matrices, where PoSaV has been shown to persist on leafy greens under refrigerated conditions (Esseili et al. 2015a, 2015b). Notably, Esseili et al. (2015a) evaluated virus stability in sterile water at RT using ultracentrifuged and ultrafiltered virus preparations. In that study, MNV remained stable over 28 days with no significant reduction in infectivity in either preparation, whereas PoSaV exhibited a significant decrease in infectious titres on day 28 compared to day 7. These results suggest lower stability of PoSaV compared to MNV under simplified aqueous conditions. The present study extends these observations to bottled water, where PoSaV exhibited a substantially faster inactivation rate than MNV and RRV at all tested temperatures. To our knowledge, infectivity-based persistence data for PoSaV in bottled water have not been previously reported. The greater difference observed here may be partly explained by differences in initial viral titres and experimental matrices; for example, PoSaV titres were at similar level as MNV (~ 6.0 log_10_ TCID_50_/mL) and thus higher in the study by Esseili et al. (2015a) than in the present study. Nevertheless, both studies consistently indicate that PoSaV is less stable than MNV in aqueous environments. These findings obtained using surrogates help estimate food safety risks if human enteric viruses contaminate bottled water, as bottles are often stored under similar temperature conditions during transport, retail display, and household storage as in this study. Further studies are needed to elucidate the mechanisms underlying reduced stability of PoSaV and to assess its persistence in more complex environmental waters.

The initial virus concentrations used in this study were higher than those typically expected in bottled water contamination events. However, high titres are commonly applied in experimental studies to observe a 3-log reduction in the viral load or allow accurate quantification of viral inactivation over extended periods (Arthur & Gibson, 2015; Bozkurt et al., 2015). Moreover, human faecal samples may contain extremely high viral loads (up to 10^9^ genome copies/g), indicating that elevated contamination levels can occur in source waters under certain conditions (Atmar et al., 2008). Virus aggregation has been suggested as a factor that may affect the apparent inactivation kinetics of viruses at high concentrations. Viruses within aggregates may be partially shielded from external inactivation processes, leading to slower apparent inactivation rates compared to dispersed virions (Grant, 1995; Mattle et al., 2011). However, these findings have primarily been established under disinfection conditions and cannot be directly applied to long-term virus survival in bottled water. Maeda et al. (1998) explored the effects of varying initial concentrations of white spot syndrome virus on its survival in water, demonstrating that viruses at higher initial concentrations persisted for extended periods. Consequently, future studies using environmentally relevant initial virus concentrations are necessary to assess whether virus concentration influences long-term persistence in bottled water. Accordingly, the results presented here should be regarded as representing a conservative, worst-case scenario.

This study has several limitations that should be considered when interpreting the results. The exact viral inactivation times could not be precisely determined because sampling was conducted at relatively wide intervals. Furthermore, surrogate animal viruses with similar morphological characteristics were used as models in this study. Although these surrogate viruses are widely used experimental models, their persistence and inactivation characteristics may not fully reflect those of the corresponding human pathogenic viruses (Richards, 2012). Therefore, direct comparisons between surrogate and human enteric viruses are needed to better establish how closely their persistence and inactivation behaviours correspond under relevant environmental conditions. These surrogate viruses serve as models and do not represent direct contamination scenarios, but they provide practical systems to estimate the persistence of human enteric viruses under controlled conditions. Additionally, the preparation and initial titers of the tested virus stocks were not identical, which somewhat limits direct comparability. Measuring the pH at each sampling point would also have been revealing. Otherwise, the strength of this study is that all viruses were evaluated under identical experimental conditions, including the same water matrix and temperature settings.

Future studies should also examine the effectiveness of water treatment processes, especially UV disinfection, against infectious enteric viruses and their surrogates under conditions that closely reflect those used in bottled water production. UV treatment is commonly applied during water production and is generally regarded effective for virus inactivation in low-turbidity waters (Hijnen et al., 2006). Furthermore, future research should extend these findings to more complex and diverse matrices, including food systems such as fresh produce and fruit-based beverages, where physicochemical properties and microbial communities may substantially influence viral persistence. In addition, a broader range of enteric viruses and genotype strains should be investigated, as survival characteristics may vary considerably even within closely related viruses. From a risk assessment perspective, identifying the most resistant viruses under relevant conditions would be important, as safety regulations should account for worst-case scenarios to ensure adequate public health protection.

## Conclusions

This study demonstrates that MNV, RRV, and PoSaV can persist in bottled drinking water over extended periods, highlighting their notable environmental stability. Temperature conditions used are directly relevant to food safety, as bottled water may be exposed to similar environments during distribution and storage. The results indicate that the infectivity of the tested enteric virus surrogates decreased gradually over time rather than undergoing rapid inactivation under the tested storage conditions. MNV and RRV remained infectious in bottled water for at least one year at 4 °C. The viral inactivation rate increased with temperature. Differences observed among the viruses further emphasise that survival characteristics may vary depending on viral structure and properties, as well as environmental conditions, including the matrix. These findings provide valuable insights into the persistence of enteric viruses in drinking water systems and underscore the importance of clean raw water, effective water treatment, and monitoring strategies for bottled drinking water. Future studies incorporating human pathogenic viruses and more complex environmental conditions are warranted to better assess real-world risks and improve food safety and public health protection.

## Data Availability

No datasets were generated or analysed during the current study.
